# High BRAF^V600E^ mutation frequency in Chinese patients with papillary thyroid carcinoma increases diagnostic efficacy in cytologically indeterminate thyroid nodules

**DOI:** 10.1097/MD.0000000000016343

**Published:** 2019-07-12

**Authors:** Xing-Jia Li, Xiao-dong Mao, Guo-fang Chen, Qi-feng Wang, Xiao-qiu Chu, Xin Hu, Wen-bo Ding, Zheng Zeng, Jian-hua Wang, Shu-hang Xu, Chao Liu

**Affiliations:** a Department of Endocrinology, Affiliated Hospital of Integrated Traditional Chinese and Western Medicine, Nanjing University of Chinese Medicine; b Key Laboratory of TCM Syndrome & Treatment of Yingbing of State Administration of Traditional Chinese Medicine, Jiangsu Province Academy of Traditional Chinese Medicine; c Department of Ultrasound; d Department of Pathology; e Department of General Surgery, Affiliated Hospital of Integrated Traditional Chinese and Western Medicine, Nanjing University of Chinese Medicine (Jiangsu Province Academy of Traditional Chinese Medicine), Nanjing, Jiangsu, China.

**Keywords:** BRAF^V600E^ mutation, indeterminate cytology, The Bethesda system for reporting thyroid cytopathology, thyroid nodule

## Abstract

To estimate the BRAFV600E mutation frequency in Chinese patients with papillary thyroid carcinoma (PTC), and the diagnostic value of BRAFV600E mutation status in thyroid nodules with indeterminate TBSRTC categories.

A total of 4875 consecutive samples for thyroid ultrasound-guided fine-needle aspiration cytology (FNAC) and BRAF^V600E^ mutation analysis were collected from patients at Jiangsu Province Hospital on Integration of Chinese and Western Medicine. Among all the cases, 314 underwent thyroidectomy. According to TBSRTC categories, FNAC was performed for a preoperative diagnosis. ROC of the subject was constructed to evaluate the diagnostic value of these 2 methods and their combination.

BRAF^V600E^ mutation in FNAC of thyroid nodules occurred in 2796 samples (57.35%). Of 353 nodule samples from 314 patients with thyroid operation, 333 were pathologically diagnosed as PTC. Of these PTC patients, 292 (87.69%) were found to have BRAF^V600E^ mutation in their preoperative FNAC. In 175 cytologically indeterminate thyroid nodules, BRAF^V600E^ mutation identified 88% of PTC. According to ROC data, BRAF^V600E^ mutation testing had an obviously higher sensitivity (87.69%) and specificity (100.00%) than TBSRTC. Combining BRAF^V600E^ mutation testing and TBSRTC achieved the largest AUC (0.954). For 41 PTC with a negative BRAF^V600E^ mutation in preoperative evaluation, the repeated BRAF^V600E^ mutation testing found out 12 samples with BRAF^V600E^ mutation. The true BRAF^V600E^ mutation rate of Chinese PTC patients was 91.29%.

Chinese patients with PTC have a higher frequency of BRAF^V600E^ mutation. The BRAF^V600E^ mutation testing affords a high diagnostic value in thyroid nodules with indeterminate cytology.

## 1 Introduction

Thyroid nodule has become a frequently-occurring clinical disease worldwide.^[1]^ Currently, serum thyrotropin measurement, neck ultrasound, and fine-needle aspiration cytology (FNAC) still remain the cornerstones for the differentiation of thyroid nodule. For nodules with suspicious features of malignancy by ultrasound, FNAC is usually supplemented to decide their nature and direct the following treatment. The Bethesda system for reporting thyroid cytopathology (TBSRTC) has been widely used. Ever since its birth in October 2007, TBSRTC has been recommended twice by American Thyroid Association (ATA) management guidelines for patients with thyroid nodules and differentiated thyroid cancer, in 2009^[2]^ and 2016,^[1]^ respectively. These recommendations provided a strategic methodology for thyroid nodule management. When FNAC is hard to confirm the diagnosis, molecular detection on BRAF^V600E^ mutation, or other abnormalities may be effective ancillary testing to identify cancerous nodules. The present study was designed to estimate the BRAF^V600E^ mutation frequency in Chinese patient with papillary thyroid carcinoma (PTC), and the diagnostic value of BRAF^V600E^ mutation status in thyroid nodules with indeterminate TBSRTC categories.

## 2 Materials and methods

### 2.1 Patients and samples

A total of 4875 consecutive samples for thyroid ultrasound-guided FNAC and BRAF^V600E^ mutation testing were collected from patients at Affiliated Hospital of Integrated Traditional Chinese and Western Medicine, Nanjing University of Chinese Medicine. Among those samples, 353 nodules of 314 patients (70 males and 244 females, ages 10 to 66 years, mean of 43.29 ± 12.05 years) were examined. All the patients have voluntarily signed the consent forms for confirmation, and the research was approved by the ethics committee of the hospital (2017LWKYZ004).

### 2.2 Find-needle aspirate and TBSRTC categories

Using locally available disposable syringes (5 ml and 10 ml) and TWLB syringe needles (0.8 mm × 38.0 mm), FNA guided by ultrasound (HITACHI HIVISION Preirus) was implemented on each patient. FNA samples were manually smeared, fixed with 95% ethanol, and HE-stained. According to TBSRTC^[2]^, the cytology of each nodule were reported as 1 of 6 categories: I, nondiagnostic or unsatisfactory (ND/UNS); II, benign (B); III, atypia of undetermined significance or follicular lesion of undetermined significance (AUS/FLUS); IV, follicular neoplasm or suspicious for a follicular neoplasm (FN/SFN); V, suspicious for malignancy (SM); VI, malignancy (M).

### 2.3 BRAF^V600E^ mutation testing

For FNA samples or cancer tissues obtained after thyroidectomy, the genomic DNA was extracted with nucleic acid extraction kits (AmoyDx Diagnostics, Co., Ltd., Xiamen, China). Then the fresh DNA was diluted to 0.4–1 ng/μl, and paraffin-fixed DNA to 2–3 ng/μl. According to the instructions of human BRAF^V600E^ mutation testing kit (AmoyDx Diagnostics, Co., Ltd., Xiamen, China), the reagent was prepared in the following way: every 35 μl reagent was mixed with 0.4 μl Taq polymerase; then the mixture was transferred into PCR tubes, closed, centrifugated, and placed into a PCR instrument (Life Technologies, Quant Studio Dx) for the process of quantitative PCR: stage I (95°C, 5 minutes, 1 cycle); stage II (95°C and 25 seconds, 64°C and 20 seconds, 72°C and 20 seconds, 15 cycles); stage III (93°C and 25 seconds, 60°C and 35 seconds , 72°C and 20 seconds , 31 cycles). In the thermal cycle at 60°C at stage III, FAM, and VIC signals were collected for real-time PCR analysis. The data were saved. After the reaction, the results were reported according to the value of FAM signals: <28 was considered positive and >28 negative.

### 2.4 Statistical analysis

SPSS 22.0 was used for statistical analysis. All quantitative data were shown as mean ± standard deviation. The best diagnostic cutoff values of TBSRTC and BRAF^V600E^ mutation testing were decided by receiver operating characteristic (ROC) curve. When 2 methods were combined to make a diagnosis, any positive result from 1 method indicated the nodule was considered as positivity. Sensitivity, specificity, positive predicative value (PPV), and negative predicative value (NPV) were all measured. ROC was drawn, and area under curve (AUC) was calculated. All these indexes were unified to make a diagnosis. *P* < .05 was considered to be statistically significant.

## 3 Results

### 3.1 Demographic data and histological results

A total of 4875 samples were studied. Preoperative FNAC and BRAF^V600E^ mutation testing provided the complete measurements of 353 nodules in 314 patients (70 males and 244 females, from ages 10 to 66 years old, with average age of 43.29 ± 12.05 years). Of 353 nodules, 333 nodules were postoperatively diagnosed as PTC. The other 20 nodules included 8 with nodular goiter, 4 with lymphocytic thyroiditis, 5 with thyroid follicular adenoma, 1 with atypical adenoma, 1 with collagen fibrous proliferation accompanied with atypical follicular epithelium hyperplasia, and 1 in normal condition.

### 3.2 TBSRTC categories of preoperative nodules

Totally, 4875 FNA-obtained nodules were classified into 6 categories: ND/UNS (1495, 30.67%), B (366, 7.51%), AUS/FLUS (359, 7.36%), FN/SFN (132, 2.71%), SM (1467, 30.09%), and M (1056, 21.66%) (Table 1). Among them, 353 nodules of 314 thyroidectomized patients were pathologically examined (Table 2). The results showed that 53, 3, 24, 4, 152, and 117 nodules could be listed into the above 6 categories. The malignancy rate of these 6 categories was 73.58% (39/53), 66.67% (2/3), 95.83% (23/24), 25.00% (1/4), 99.34% (151/152), 100.00% (117/117), respectively.

**Table 1:**
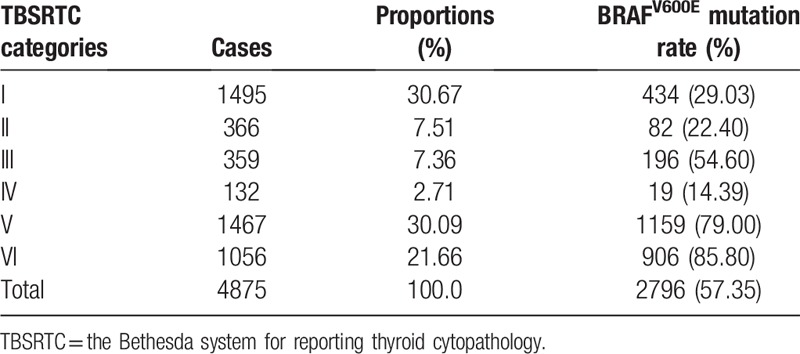
Distribution of TBSRTC categories, BRAF^V600E^mutation in 4875 thyroid nodules.

**Table 2:**
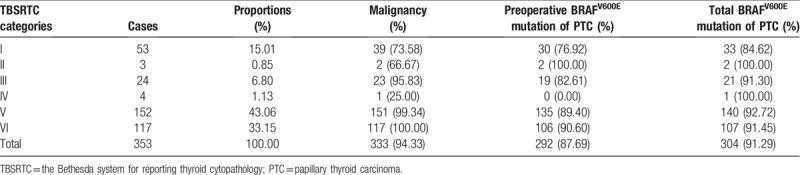
Distribution of TBSRTC categories, BRAF^V600E^mutation and postoperatively histopathological results in 314 patients.

### 3.3 Results of preoperative BRAF^V600E^mutation testing

BRAF^V600E^ mutation testing was performed in 4875 FNA-obtained samples. Among them, 2796 nodules (57.35%) were found to have gene mutation. The BRAF^V600E^mutation rate in the 6 categories of nodules was 29.03%, 22.40%, 54.60%, 14.39%, 79.00%, and 85.80%, respectively (Table 1).

Among 353 thyroidectomized nodules, 292 nodules had BRAF^V600E^ mutation before surgery, and were diagnosed as PTC by postoperative pathology. The BRAF^V600E^ mutation rate in the 6 categories of nodules was 56.60%, 66.67%, 79.17%, 0.00%, 88.82%, and 90.60%, respectively (Table 2). Three indeterminate categories were further analyzed. The mutation was not found in 4 FN/SFN nodules, of which 3 were benign and 1 was malignant. The mutation rate was higher than 80% in nodules with AUS/FLUS. In addition, samples of 41 PTC without BRAF^V600E^ mutation in preoperative measurement were paraffin-embedded for a repeated BRAF^V600E^ mutation testing. Finally, 12 PTC were found to have BRAF^V600E^ mutation.

### 3.4 Comparison of the diagnostic value among TBSRTC, BRAF^V600E^ mutation testing, and their combination

In present study, ROC statistical analysis showed that the sensitivity, specificity of TBSRTC for the diagnosis of thyroid nodules was 80.48% and 95.00%. However, the sensitivity and specificity of BRAF^V600E^ mutation detection were significantly higher than TBSRTC (87.69% and 100.00%, respectively). There was no significant difference between the AUC of TBSRTC and BRAF^V600E^ mutation detection (0.895 vs 0.938, *P* = .0752). The combination of TBSRTC and BRAF^V600E^ mutation testing achieved the highest AUC (0.954), which was significantly higher than TBSRTC (*P* = .0009) but not (*P* = .5515). The sensitivity of the combination increased to 95.80%, and the specificity was the same as the single use of TBSRTC (Table 3).

**Table 3:**
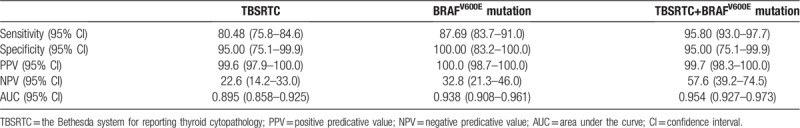
Comparison of the diagnostic value of TBCRTC and BRAF^V600E^ analysis.

## 4 Discussion

FNAC is the most accurate and cost-effective tool to evaluate thyroid nodules suspicious for malignancy with ultrasound. TBSRTC, the major landmark of thyroid cytology, is the creation of a uniform system for reporting thyroid cytopathology. Due to 6 recognized diagnostic categories with an incremental risk of malignancy, TBSRTC standardized the cytological diagnosis and increased its diagnostic yield. A number of clinical studies has demonstrated that this system did not clearly answer for the heterogeneous group of nodules with indeterminate cytology, including AUS/FLUS, FN/SFN, and SM. The genetic marker BRAF V600E mutation, the most robust oncogene in PTC, has drawn particular attention and been widely tested.

In present study, we analyzed the results of FNAC and BRAF^V600E^ mutation testing in a large population. The analysis on thyroidectomized patients clarified that the malignancy risk of nodules in TBSRTC I, II, III apparently higher than those described by ATA guidelines.^[1]^ Nodules with indeterminate cytology were also found to have a high malignancy risk in our study. The molecular testing were recommended to improve the diagnostic efficacy for these nodules.^[2,3]^ The preoperative detection of BRAF^V600E^ mutation can identify most of malignancy in AUS/FLUS nodules. Only a few of patients with nodules were categorized as FN/SFN were surgically treated. Among them, the malignancy rate was 25.00% and no BRAF^V600E^ mutation were detected preoperatively. Previous researches demonstrated that BRAF^V600E^ mutation arises in tall cell variant and conventional PTC with more possibilities, while it seldom occurs in follicular variant and follicular thyroid neoplasm.^[4]^ Also, the absence of commercial molecular detection methods in China remains an obstacle to implement the accurate diagnose for FN/SFN nodules.

In this research, totally 87.69% of PTC were found to have BRAF^V600E^ mutation, and all the nodules with BRAF^V600E^ mutation were confirmed to be PTCs. The mutation rate is much close to the research of Zhang et al in Nanjing^[5]^ and Guo et al in Beijing,^[6]^ but higher than the date from some other Chinese researchers.^[7]^ Taken them together, the mutation rate of BRAFV600E in Chinese PTC patients seems to be higher than that in the studies of Kim et al, Kim et al and Kim et al from South Korea,^[8–10]^ Xing et al from the United states,^[11]^ and Beisa et al from Lithuania.^[12]^ A reasonable answer for the difference of mutation rate of BRAF^V600E^ in different regions is still required. Guan et al^[7]^ has found that the prevalence of BRAF mutation was significantly higher in any of the regions with high iodine content than any of the regions with normal iodine content (69% vs 53%). Our previous study demonstrated that Nanjing were regarded as the region with more than adequate iodine intake according to the median urine iodine concentration of school-age children (median 282 μg/L),^[13]^ which is much higher than the normal.^[14]^

Surprisingly, among 41 PTC which were preoperatively negative to BRAF^V600E^ mutation, the repeated BRAF^V600E^ mutation testing found 12 mutated PTC. Taken them together, the true BRAF^V600E^ mutation rate of PTC was 91.29%, indicating a different genotype of PTC in Chinese population. The false negative results may originate from the inappropriate sample, or variants of PTC.^[15]^

The sensitivity (80.48%), specificity (95.00%), PPV (99.6%), and NPV (22.6%) indicated the favorable diagnostic value of TBSRTC. *BRAF* is a gene most likely to mutate in PTC. More than 90% of BRAFs mutations result in an amino acid substitution at position 600 in *BRAF*, from a *valine* (V) to a glutamic acid (E). In our research, both the sensitivity and specificity of BRAF^V600E^ mutation testing rise up above those of TBSRTC. Their combination can significantly increase the NPV of cytological diagnosis, and rule out indeterminate nodules (especially those of category NON, B, and AUS/FLUS) caused by small sample size, low-quality sections, and scant experience of pathologists.

In conclusion, Chinese patients with PTC have a high BRAF mutation frequency, and the BRAF^V600E^ mutation testing shows a higher sensitivity and specificity. Combining TBSRTC and BRAF^V600E^ mutation testing can improve the diagnostic sensitivity and reduce the rate of indeterminate cytological diagnosis.

## Author contributions

**Data curation:** Shuhang Xu.

**Formal analysis:** Xing-Jia Li, Xiao-dong Mao, Shuhang Xu.

**Funding acquisition:** Guo-fang Chen, Shuhang Xu.

**Investigation:** Shuhang Xu.

**Methodology:** Xing-Jia Li, Xiao-dong Mao, Qi-feng Wang, Xiao-qiu Chu, Xin Hu, Shuhang Xu.

**Project administration:** Shuhang Xu.

**Resources:** Wen-bo Ding, Zheng Zeng, Jian-hua Wang.

**Supervision:** Chao Liu.

**Writing – original draft:** Xing-Jia Li.

**Writing – review & editing:** Shuhang Xu.

Shuhang Xu orcid: 0000-0002-8619-5376.
